# Placental and Umbilical Cord Anomalies Diagnosed by Two- and Three-Dimensional Ultrasound

**DOI:** 10.3390/diagnostics12112810

**Published:** 2022-11-16

**Authors:** Eduardo Félix Martins Santana, Renata Gomes Castello, Giuseppe Rizzo, Gianpaolo Grisolia, Edward Araujo Júnior, Heron Werner, Mario Lituania, Gabriele Tonni

**Affiliations:** 1 Medical Course, Albert Einstein Medical School, Sao Paulo-SP 05652-900, Brazil; 2 Fetal Medicine Unit, Albert Einstein Hospital, Sao Paulo-SP 05652-900, Brazil; 3 Department of Obstetrics and Gynecology, Policlinic Hospital, Tot Vergata Foundation, Tor Vergata University, 00133 Rome, Italy; 4 Department of Obstetrics and Gynecology, Carlo Poma Hospital, 46100 ASST Mantua, Italy; 5 Department of Obstetrics, Paulista School of Medicine—Federal University of Sao Paulo (EPM-UNIFESP), Sao Paulo-SP 04021-001, Brazil; 6 Medical Course, Municipal University of Sao Caetano do Sul (UCS), Bela Vista Campus, Sao Paulo-SP 09550-051, Brazil; 7 Centro Diagnostico Por Imagem, Rio de Janeiro 24070-035, Brazil; 8 Preconceptional and Prenatal Pathophysiology, Department of Obstetrics and Gynecology, E.O. Ospedali Galliera, 16128 Genoa, Italy; 9 Department of Obstetrics and Neonatology, and Researcher, Istituto di Ricovero e Cura a Carattere Scientifico (IRCCS/AUSL), 42122 Reggio Emilia, Italy; Tonni.Gabriele@ausl.re.it

**Keywords:** placental pathology, umbilical cord pathology, two-dimensional ultrasound, three-dimensional ultrasound, perinatal outcome, autopsy

## Abstract

The aim of this review is to present a wide spectrum of placental and umbilical cord pathologies affecting the pregnancy. Placental and umbilical cord anomalies are highly associated with high-risk pregnancies and may jeopardize fetal well-being in utero as well as causing a predisposition towards poor perinatal outcome with increased fetal and neonatal mortality and morbidity. The permanent, computerized perinatology databases of different international centers have been searched and investigated to fulfil the aim of this manuscript. An extended gallery of prenatal imaging with autopsy correlation in specific cases will help to provide readers with a useful iconographic tool and will assist with the understanding and definition of this critical obstetrical and perinatological issue.

## 1. Introduction

The sonographic evaluation of the placenta and umbilical cord are often underestimated by ultrasound professionals and obstetricians [1]. However, the anomalies of these fetal compartments can be associated with important prognostic implications for perinatal morbidity and mortality [1]. Placental abnormalities are responsible for a stillbirth rate ranging from 11.2% to 64.9% of stillbirths, when ischemic placental diseases [2] and abnormal placentation are associated with fetal growth abnormalities linked or not to stillbirth [3]. Umbilical cord anomalies are responsible for 28% of all stillbirth cases beyond 32 week’s gestation, in addition to other adverse pregnancy outcomes, such as birth asphyxia and emergency Cesarean section [4,5] 

In this article review, we aim to demonstrate the main prenatal diagnoses of placental and umbilical cord anomalies by two- and three-dimensional ultrasound and fetal magnetic resonance imaging (MRI) with autopsy correlation in specific cases.

## 2. Materials and Methods

The permanent, computerized perinatology databases of different international centers have been investigated. A search was conducted for placental and umbilical cord pathology, intrauterine outcome, diagnostic procedures and neonatal outcome. When indicated, prenatal imaging diagnosis was compared with autopsy examination. All ultrasound imaging was obtained from mothers undergoing standard screening examinations or from extended ultrasound and fetal MRI (magnetic resonance imaging) in targeted cases, in agreement with national and international guidelines for the use of ultrasound and MRI in obstetrics population and in agreement with the best gold standard of care. For these reasons, no Ethics Committee or Institutional Review Board approval was necessary.

## 3. Results

### 3.1. Placental Anomalies

#### 3.1.1. Placenta Accreta Spectrum 

**Figure 1:**
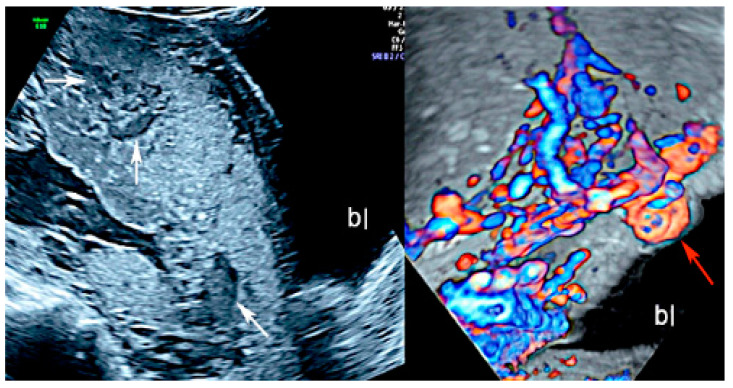
Two-dimensional ultrasound imaging showing PAS (placenta accreta spectrum, placenta increta type): longitudinal scan in a patient at 24 week’s gestation shows multiple hypoechoic areas (lacunae) (white arrows). Color Doppler ultrasound shows increased vascularity and the bulging of the placenta increta (red arrow); (bl): bladder.

Placenta accreta spectrum (PAS) is the general term used to describe abnormalities related to the adherence of the placental trophoblast to the uterine endometrium and myometrium [6,7,8,9,10]. The PAS includes three types of anomalies: placenta accreta, which is the excessively firm attachment of the placenta to the endometrium decidua; placenta increta, which is characterized by the invasion of the trophoblast into the myometrium; and placenta percreta, in which the myometrium, serosa and even surrounding structures are invaded by placental peptides [6,7,8,9,10]. Adherent placenta is histologically caused by the trophoblast’s invasion of the spongiosa layer of the decidua, possibly because of a damaged or deficient Nitabuch’s layer [7,8,10], and its incidence has risen due to the increase of surgical procedures, such as myomectomy, Cesarean section, endometrial ablation and uterine curettage [6,7,8,9]. PAS may be associated with an increase in maternal mortality, mainly due to massive bleeding and its consequences after attempting to separate the placenta from the myometrium but also after uterine rupture [6,11]. Several studies have documented that ultrasonography performed by an experienced operator has the sensitivity and specificity in the grey scale modality as high as 90%, and sonographic markers associated with PAS are multiple vascular lacunae, the loss of the normal hypoechoic retroplacental zone in the myometrium, the abnormality of the uterine serosa-bladder interface, the thinning of the retroplacental myometrium, the bulging of the lower uterine segment, and increased placental vascularity on color Doppler [6,9,10,11,12,13,14,15]. (Figure 1).

**Figure 2:**
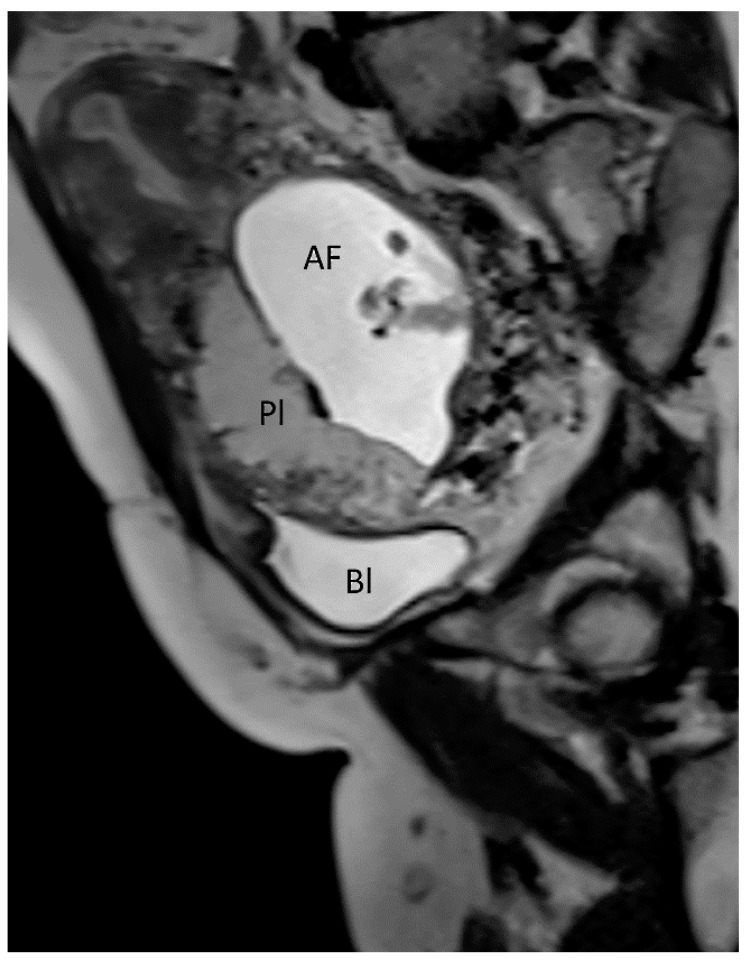
Fetal MRI (13 weeks, sagittal T2) showing an early diagnosis in a case of PAS (placenta accreta, type) (Legend: AF, amniotic fluid; Bl, bladder; Pl, placenta).

The presence of ultrasound marker as the “rail sign”, which consists in two parallel neo vascularizations on color Doppler ultrasound, originating between the uterovesical junction and the bladder mucosa, is related with an increased risk of placenta increta or percreta. Therefore, higher risks of adverse clinical outcomes, such as hysterectomy, blood transfusion, admission to the intensive care unit and bladder invasion may occur [16]. Prenatal diagnosis is essential to prevent the morbidities associated with this pathology although two-thirds of patients do not have a correct diagnosis before delivery [12]. It has been argued whether MRI may improve the detection rate compared with ultrasound in the diagnosis of PAS [12,13,17]. MRI has higher costs and limited access at local facilities; for these reasons, MRI is still considered a second-line diagnostic investigation [12,13,18]. MRI has shown an excellent interobserver agreement in the identification of presence and depth of PAS disorders, especially when the placenta is located posteriorly or laterally or in case of increased maternal body mass index (BMI), as well as in cases of the suspected involvement of surrounding organs [12,13,18]. A prospective study by Finazzo et al. [18] reported only moderate agreement in the identification of the topography of placental invasion through MRI evaluation. The radiologic clusters of PAS are the following: abnormal placental vascularization, the disruption of tri-layered appearance of the myometrium, the presence of a lobulated myometrium-placental margin, the disruption of the smooth outer uterine contour due to the outward bulging of the placenta, the presence of intraplacental dark bands and the visualization of direct invasion of adjacent organs [19,20] (Figure 2).

#### 3.1.2. Placenta Praevia

**Figure 3:**
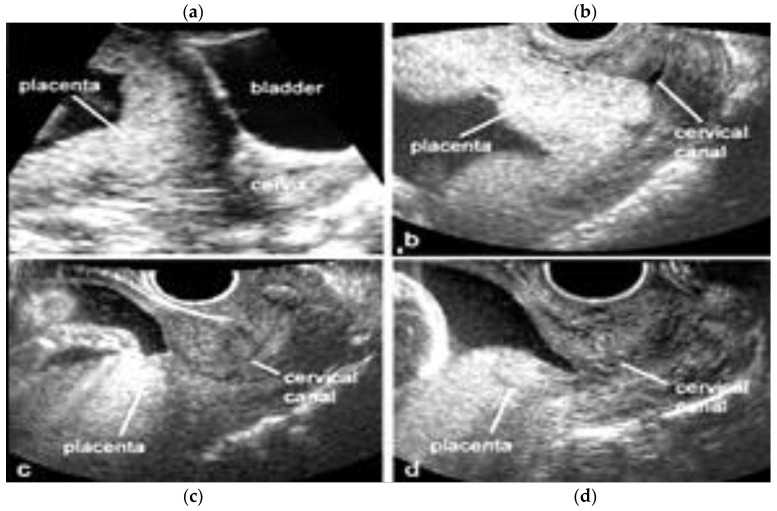
Transvaginal two-dimensional ultrasound showing placenta praevia major (upper panel, **a**,**b**) and a placenta praevia minor (lower panel, **c**,**d**).

**Figure 4:**
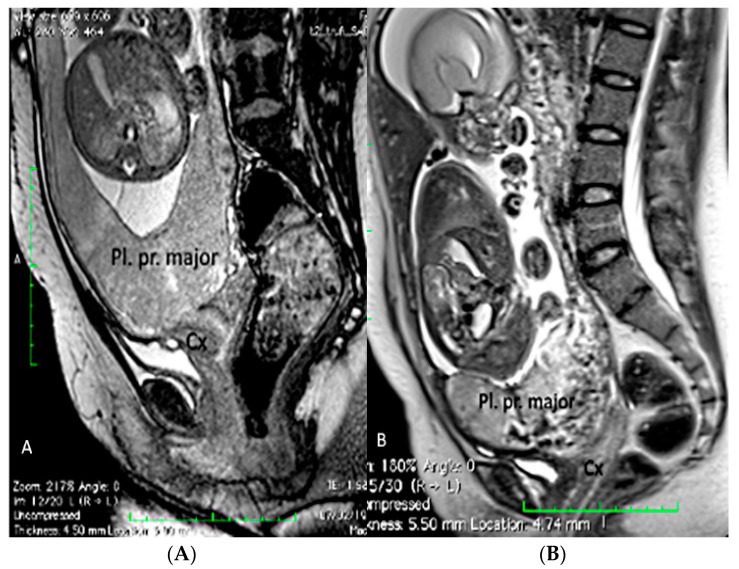
Fetal MRI (sagittal T2) performed at 30 weeks and 26 week’s gestation in two different fetuses (**A**,**B**), demonstrating a placenta praevia major and accretism. (Legend: Pl: placenta; Cx: cervix).

Placenta praevia is a condition in which the placenta develops within the lower uterine segment and overlies the cervix [7]. Previous terminology, such as symmetric or complete, has now been replaced and the condition is now classified as placenta praevia major [7,21,22] On the other hand, if the placenta does not overlie the cervix, but its edge is located within 2.0 cm of the cervical os, it is called a low-lying placenta. This type of placenta is now defined as placenta praevia minor [7,22]. The incidence of placenta praevia is seen in around 4 to 5 of 1,000 pregnancies, and the risk factors associated with this condition are advanced maternal age, multiparity, previous placenta praevia, chronic hypertension, diabetes mellitus, smoking during pregnancy, multiple gestation and assisted reproductive technology (ART) [22]. This placental anomaly, besides being one of the major risk factors for PAS, is associated with maternal bleeding, Cesarean section and prematurity [7,22,23]. The use of transvaginal ultrasound allows for the better definition of the placental anatomy in relation to the cervix with a sensitivity of 88% and specificity of 99% [22]. In the face of placenta praevia and low-lying placenta, the gray-scale third trimester ultrasound has a great negative predictive value in detecting cases of accretism when normal hypoechogenic space is seen between the uterus and placenta and through the lack of the visualization of placental lacunae [24]. Ultrasound has shown a low sensitivity in the diagnosis of PAS in mothers with posterior placenta praevia while MRI has superior accuracy and fewer false negative results, but higher accuracy was obtained through a combination of both techniques [23,25,26] (Figure 3 and Figure 4).

#### 3.1.3. Bilobed Placenta and Succenturiate Lobe

**Figure 5:**
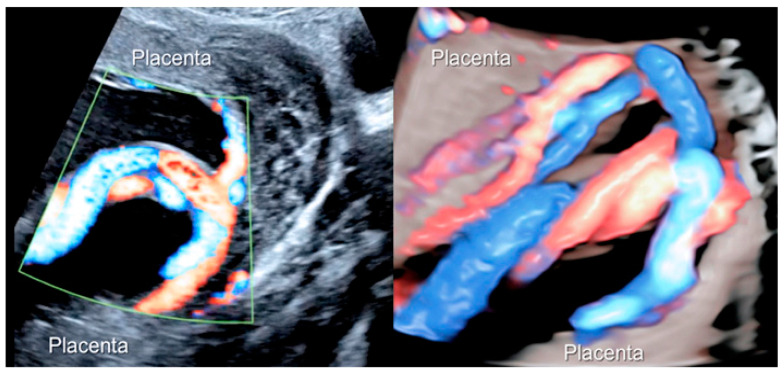
Two-dimensional color Doppler ultrasound and three-dimensional ultrasound using HD-Live™ flow showing a bilobed placenta. When an accessory lobe is detected (succenturiate) in the lower part of the uterus, careful evaluation with ultrasound for vasa praevia and velamentous cord insertion should be performed.

**Figure 6:**
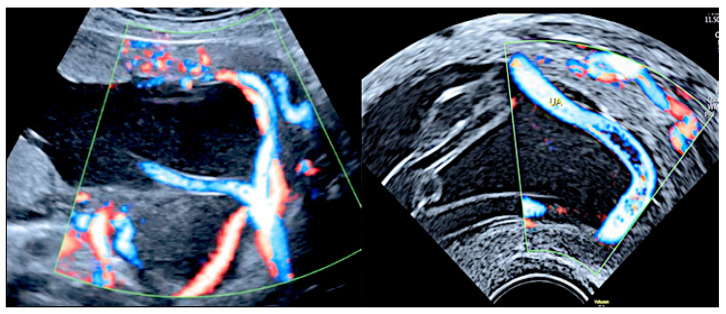
Pregnancy at 20 week’s gestation. Two-dimensional color Doppler ultrasound showing velamentous insertion in a bilobed placenta. (Legend: UA, umbilical artery).

**Figure 7:**
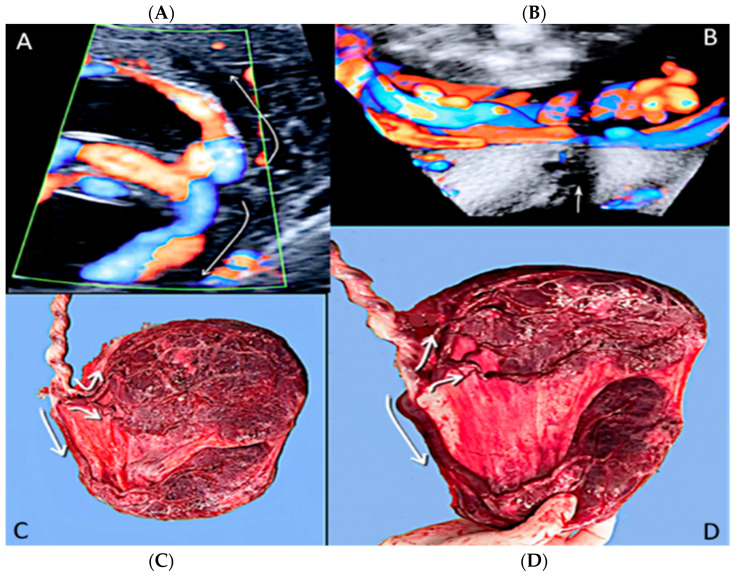
Pregnancy at 21 weeks’ gestation. Succenturiate placenta and placenta praevia Type II. Color Doppler and four-dimensional (4D) STIC with color Doppler. Low-lying placenta and accessory lobe are connected by intramembranous blood vessels, with the umbilical cord originating from the main placenta (**A**). Vasa praevia and contemporaneous cord *procidentia* are highlighted (cervical canal is indicated by a small arrow) (**B**). Pathologic correlation (**C**,**D**) showed two separate placental lobes interconnected by membranes, with blood vessels traversing the membranes (indicated by curved arrows).

Bilobed placenta is a placental morphological anomaly that refers to a placenta separated into two roughly equal-sized lobes, separated by membranes, and its incidence is around 2-8% of placentas [16,17]. The diagnosis is made via ultrasound assessment by the visualization of two separated placental discs of nearly equal size [16,17]. A succenturiate lobe is a smaller accessory placental lobe that develops in the membranes, apart from the main disc of the placenta. This condition is diagnosed in 5% of all pregnancies by ultrasound scan as a smaller separate lobe, away from the main placental lobe [16,17]. It is important to exclude the presence of connecting vessels between the main placental discs and the accessory discs, which can be achieved with the use of color Doppler ultrasound, as these two conditions are a risk factor for vasa praevia if the connecting vessel is positioned over the internal os [27,28,29,30]. The Doppler ultrasound can also differentiate a connecting vessel between the two placental lobes from amniotic band [30]. The use of MRI in the evaluation of bilobed placentas and succenturiate lobes is still scantily reported in literature, although MRI may be an additional diagnostic *armamentarium* in detecting the number and location of the placental lobes and in the evaluation of vasa praevia associated with placental anomalies [31]. Overall, the prenatal diagnosis of these conditions is of vital importance to prevent or avoid post-partum hemorrhage due to retained placental tissue [27,28,29] (Figure 5, Figure 6 and Figure 7).

#### 3.1.4. Circumvallate Placenta

**Figure 8:**
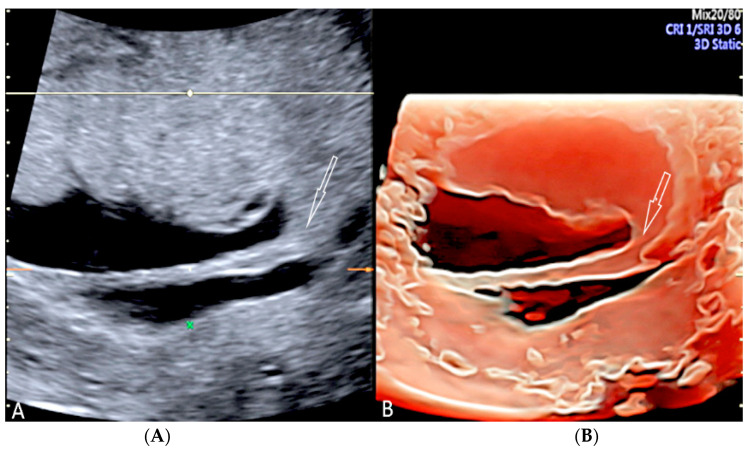
Circumvallate placenta with anterior placenta at two-dimensional ultrasound (**A**). Three-dimensional ultrasound using HD-Live Silhouette™ mode (**B**) at 20 week’s gestation shows the raised edge of the placenta as a linear band of tissue that may mimic a uterine synaechia (arrows).

**Figure 9:**
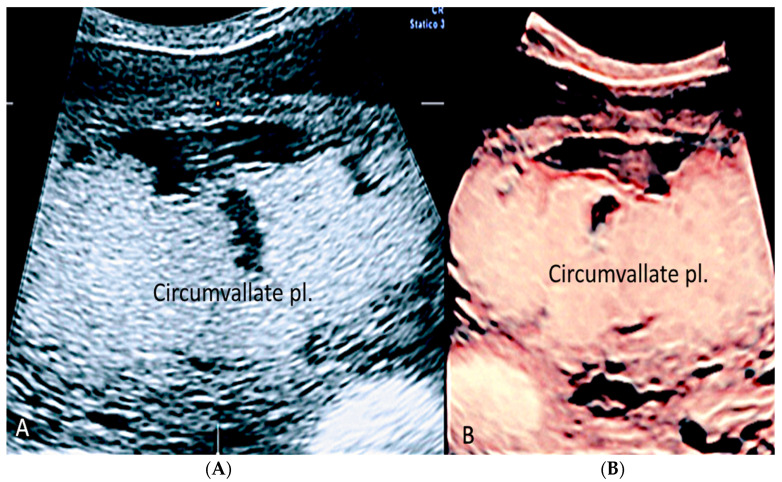
(**A**,**B**) Circumvallate placenta (pl.) with posterior placenta. Two-dimensional and three-dimensional ultrasound using HD-Live™ mode showing a “shelf-like” structure.

**Figure 10:**
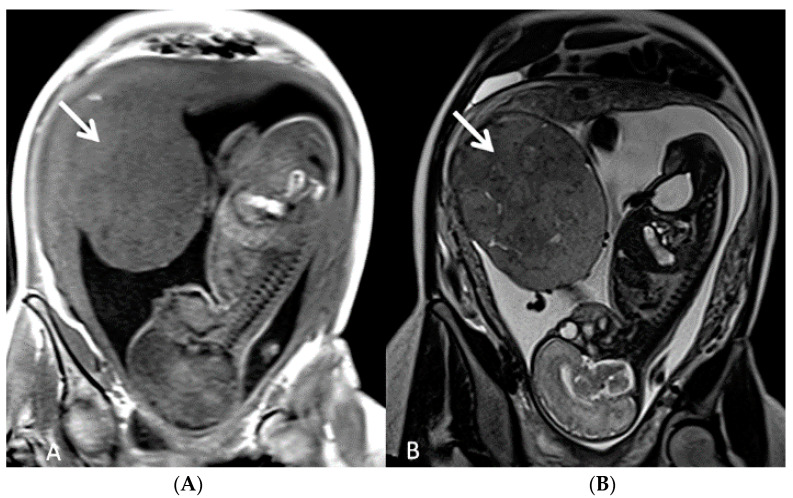
(**A**,**B**) Fetal MRI (coronal T1 and T2) in a case of circumvallate placenta at 28 week’s gestation (white arrows).

Circumvallate placenta is an abnormality in the area between the placental surfaces, in which the chorionic plate is smaller than the basal plate with protruding peripheral placental tissue [1,32,33]. It is associated with a higher risk of preterm delivery, vaginal bleeding during pregnancy, subchorionic hematoma, preterm premature rupture of membranes, placental insufficiency and placental abruption, especially when maternal serum alpha-fetoprotein is elevated (1.4 MoM) (multiple of the median) and when UtA-PI (uterine artery-pulsatility index) of 1.2 [27,32,33,34]. A sonographic diagnosis can be made during the 18–21 ultrasound week scan, when the average placental thickness is 2.74 ± 0.53 cm [33]. A raised edge of the placenta appearing as an echodense ridge and a circular depression with thick peripheral ring on the chorionic plate has been proposed by Arlicot et al. [35] as the “tire sign” and may be a useful clinical marker of circumvallate placenta when using 3D ultrasound [1,27,36]. (Figure 8, Figure 9 and Figure 10).

#### 3.1.5. Placental Mesenchymal Dysplasia

**Figure 11:**
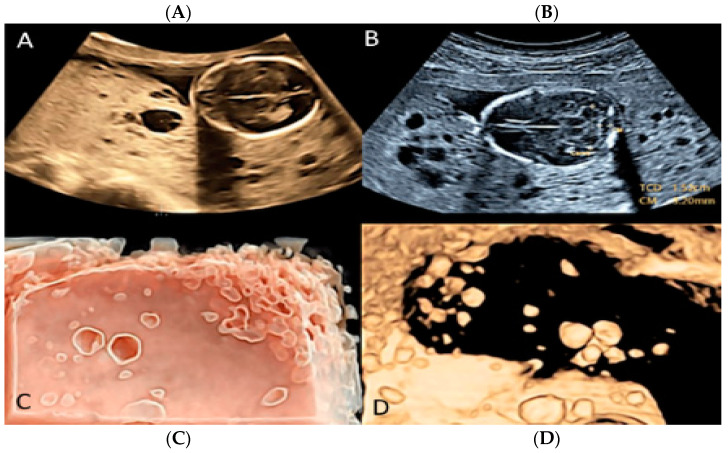
Placental with mesenchymal dysplasia at 15 weeks of gestation. Two-dimensional (**A**,**B**) and three-dimensional ultrasound (**C**,**D**) with HD-Live Silhouette™ and inversion mode displaying a characteristic large placenta with “grape-like”, multicystic changes.

Placental mesenchymal dysplasia is a rare vascular anomaly of the placenta characterized by cystic lesions, mesenchymal villous hyperplasia, and the thrombosis of the chorionic plate and stem villous vessels [27,37,38]. Sonographic diagnosis includes placentomegaly and a “grape-like” placental appearance, with multiple cystic areas, making a differential diagnosis with gestational trophoblastic disease (GST) a challenging issue [27,37,38,39]. Placental mesenchymal dysplasia may be seen to be associated with fetal growth restriction (FGR), intrauterine fetal demise (IUFD) and preterm delivery [27]. (Figure 11).

#### 3.1.6. Chorioangioma

**Figure 12:**
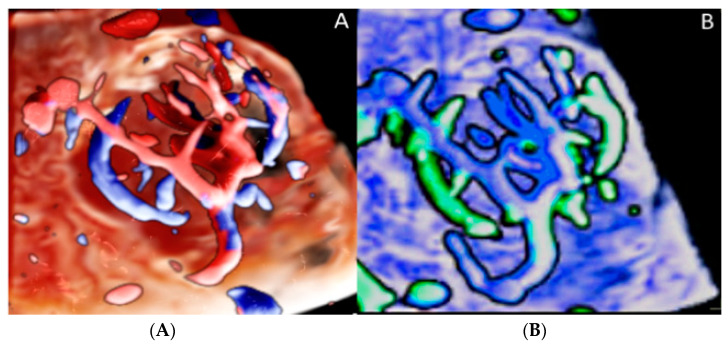
Three-dimensional ultrasound in a case of placental chorioangioma detected using Cristal Vue™ at 31 week’s gestation (**A**). Note the detail of the vascular branching (**B**).

**Figure 13:**
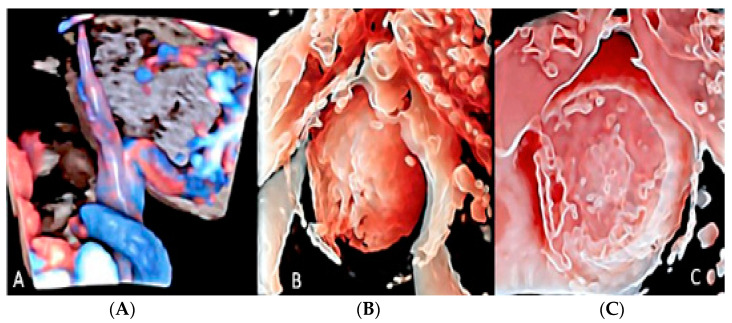
Chorioangioma. Pregnancy at 20 and 26 week’s gestation. Two-dimensional color Doppler (**A**), three-dimensional ultrasound with HD-live™ surface rendering (**B**) and with Silhouette™ effect (**C**). Chorioangioma arises from the fetal surface of the placenta, where vessels’ cords diverge before joining the placenta.

According to Fan et al. [40], chorioangioma is the most common benign non-trophoblastic tumor of the placenta, occurring in 1% of pregnancies; it is considered a vascular malformation involving the primitive placental angioblastic tissue [27,29]. The adverse outcomes associated with this condition are fetal growth restriction, hydrops, hyperdynamic circulation and perinatal morbidity when tumors progressively grow throughout pregnancy. Fetal complications may include polyhydramnios, fetal anemia and hydrops; therefore, a close follow-up is mandatory [41]. Sonographic evaluation is characterized by the visualization of hypoechoic lesions close to the insertion of the umbilical cord, appearing as rounded and well-circumscribed structures with aberrant fetal vessels nourishing the tumor [42] (Figure 12 and Figure 13).

#### 3.1.7. Membranous Placental Cysts

**Figure 14:**
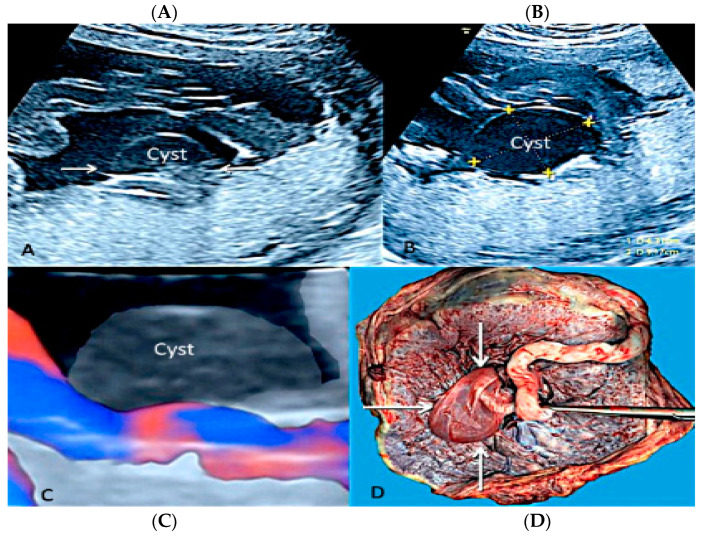
Two-dimensional (**A**,**B**) and three-dimensional ultrasound with HD flow using “glass body” mode (**C**) showing chorionic plate cyst. It depicts an anechoic thin-walled structure on the fetal surface of the placenta near the cord insertion (arrows) (**A**,**B**) without internal vascularity (**C**). Pathologic correlation (arrows) (**D**).

Membranous placental cysts, also known as “subchorionic cysts”, “chorionic cysts” or “membranous cysts” [42], are placental lesions formed by a deposit of fibrin in the subchorionic layers, forming a central cystic component. Brown et al. [43] have demonstrated that when the diameter of the cysts is greater than 4.5 cm, there is a high association with fetal growth restriction. On a grey-scale ultrasound evaluation, this type of placental disease resembles a chorioangioma and differential diagnosis is possible using color flow Doppler ultrasound, as subchorionic cysts have no flow [29] (Figure 14).

### 3.2. Umbilical Cord Anomalies

#### 3.2.1. Nuchal Cords

**Figure 15:**
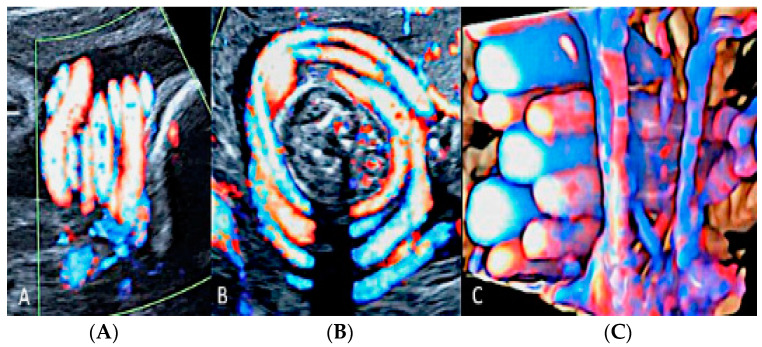
Two-dimensional ultrasound using color Doppler ultrasound and three-dimensional ultrasound with STIC color Doppler and “glass body” mode rendering with HD-Live™ flow showing multiple loops of cord around the neck in sagittal (**A**), axial (**B**) and coronal scans (**C**), resembling a “whirlpool” sign.

A nuchal cord occurs when the umbilical cord becomes entangled at 360 degrees around the fetal neck [44,45]. The prevalence of nuchal cords at birth is around 22% of all pregnancies [4], with no significant statistical association between the presence of a nuchal cord and stillbirth [4,5]. On the other hand, multiple loops of nuchal cord have been associated with abnormal fetal heart rate pattern during labor, low umbilical artery pH, an increased likelihood of Cesarean section or operative deliveries and an Apgar score < 7 at the 5th minute [4,46]. Tight nuchal loops are associated with a low Apgar score but not with an increased admission to the neonatal intensive care unit (NICU). Loose loops of nuchal cord are associated with a favorable in utero prognosis and perinatal outcome [4] (Figure 15).

#### 3.2.2. Vasa Praevia

**Figure 16:**
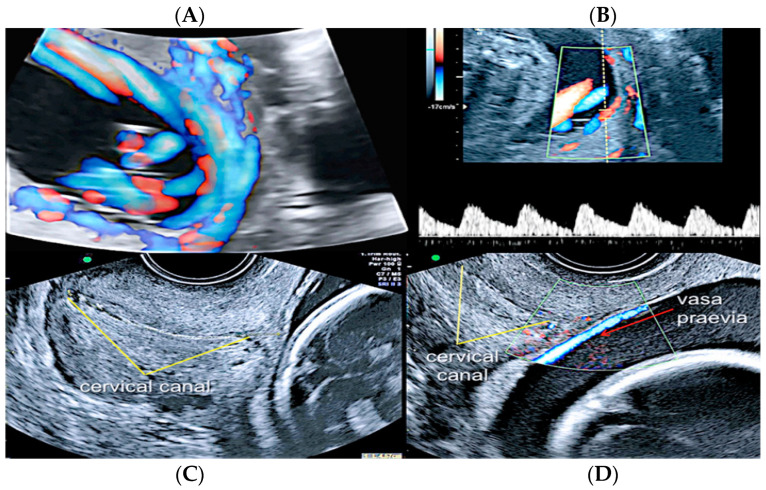
Second trimester transvaginal scan and three-dimensional ultrasound with “glass-body” rendering (**A**), Doppler ultrasound (**B**) and two-dimensional ultrasound showing the anatomical relationship between the cervical canal (yellow lines) **(C)** and the presence of vasa praevia (red arrow) (**D**).

**Figure 17:**
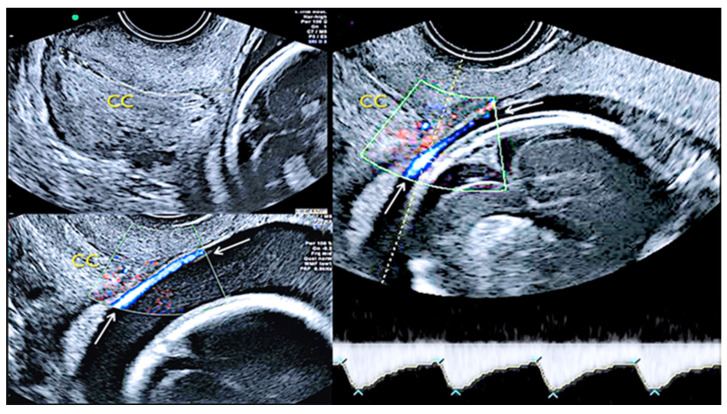
Pregnancy at 24 weeks’ gestation. Two-dimensional ultrasound, color and pulse wave (PW) spectral Doppler ultrasound allowed the detection of an arterial vessel close to the internal cervical os (vasa praevia Type III, white arrows)(Legend: CC: cervical canal).

Vasa praevia is a rare obstetrical pathology that affects 0.046% of all pregnancies, in which the fetal blood vessels are located within the membranes, not in their usual location protected by the umbilical cord or the placenta, and cover the internal cervical os [44,47]. Vasa praevia is classified into three types: Type I, when there is one single placental lobe with a velamentous cord; Type II, when the unprotected fetal vessels connect two lobes of a succenturiate lobed or bilobed placenta, near the cervix; and Type III, when the fetal vessel runs within the membranes, near the cervix, but is not associated with a velamentous cord insertion or bilobed placenta [48]. Vasa praevia has a resolution rate varying from 14% to 39% up to 28 weeks of gestational age, especially if diagnosed before 24 weeks of gestation, if not covering the internal cervical os and when not associated with placenta praevia [49]. These unprotected vessels are susceptible to rupture or compression, impairing fetal vascularization [40,46]. Perinatal survival rate is dependent on appropriate prenatal diagnosis and improves from 72% versus 97% when vasa praevia is detected antenatally [49,50]. Even if a correct prenatal diagnosis occurs, almost 70% of newborns receive NICU treatment due to the complications of prematurity [51,52,53,54]. Transvaginal ultrasound and color Doppler ultrasound are the gold standard diagnostic modalities [53]; however, due to the low incidence of this condition, there are no population screening protocols, and the screening approach is indicated only in high-risk patients [55,56], such as low-lying placenta/placenta praevia, velamentous cord insertion, ART and multiple gestations [52]. Elective Cesarean section should be planned around 35 weeks and not after 37 week’s gestation [57], and although no significant statistical differences were seen in the rate of neonatal complications between inpatient vs. outpatient management, in the outpatient group, a higher risk of an emergency Cesarean section was reported [58] (Figure 16 and Figure 17).

#### 3.2.3. Velamentous Umbilical Cord Insertion

**Figure 18:**
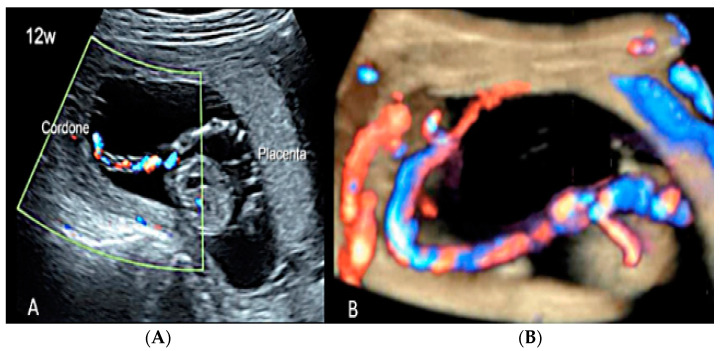
(**A**,**B**) First trimester (12 weeks) scan showing in two-dimensional- and three-dimensional color Doppler ultrasound a velamentous cord insertion (Cordone: umbilical cord).

**Figure 19:**
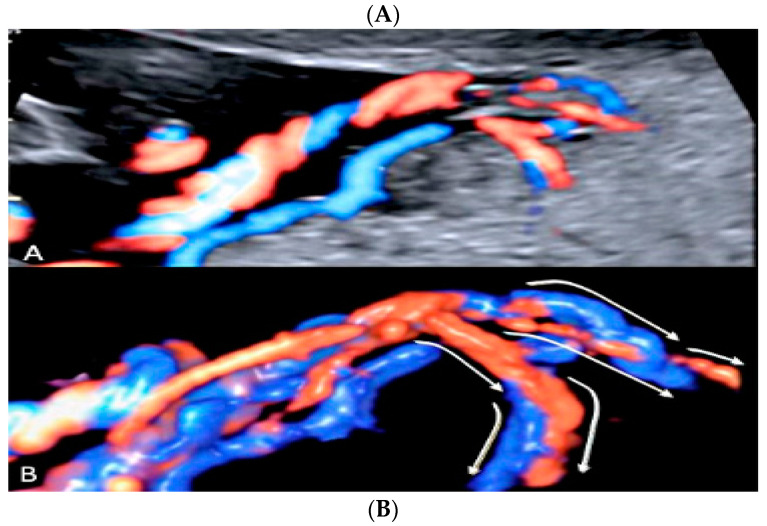
Pregnancy at 19 week’s gestation (same case of the previous figure). Two-dimensional color Doppler ultrasound (**A**) and four-dimensional color Doppler STIC volume (**B**) diagnosed velamentous cord insertion.

**Figure 20:**
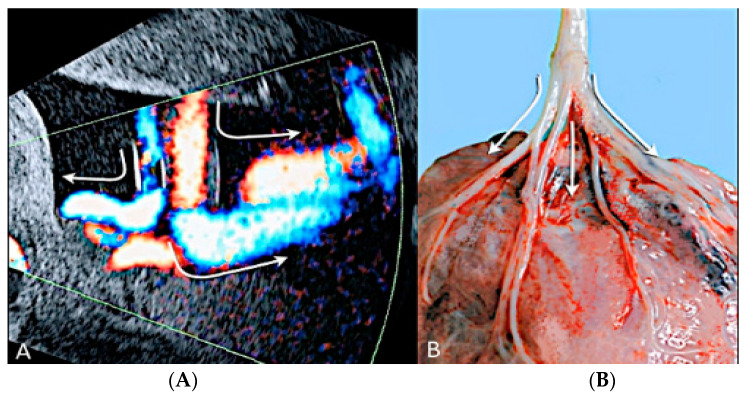
Velamentous cord insertion into the placental membranes (**A**) with direction of blood flow indicated by curved arrows and pathologic correlation (arrows) (**B**).

**Figure 21:**
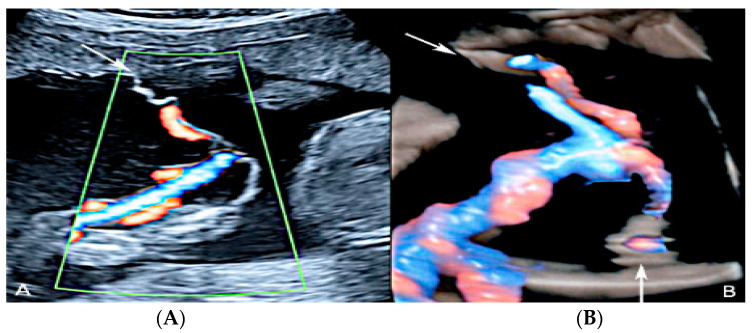
Twin pregnancy at 16 week’s gestation. Two-dimensional ultrasound (**A**) and four-dimensional STIC with color Doppler ultrasound (**B**) shows a particular velamentous insertion of the cord’s vessels that run through the intertwin membranes before joining the placenta (arrows).

**Figure 22:**
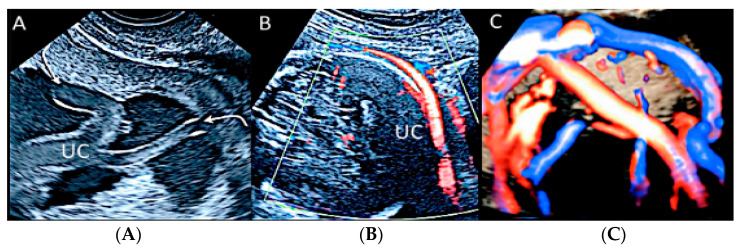
Pregnancy at 20 weeks gestation. Two-dimensional ultrasound (**A**), color Doppler (**B**) and four-dimensional STIC with color Doppler ultrasound (**C**). In velamentous insertion, the umbilical cord vessels (UC) diverge from each other, inserting into the membranes not supported by Wharton jelly, before entering the placental tissue (curved arrows, figure on the left) (**A**). The diagnosis of velamentous cord insertion is made through color Doppler ultrasound by the discovery of splayed umbilical vessels at the periphery of the placenta (**B**). It may be associated with vasa praevia (Type I) when umbilical vessels running through the fetal membranes are close to the internal cervical os (figure on the center). Four-dimensional STIC with color Doppler ultrasound (**C**) image showing the “mangrove sign” for velamentous cord insertion.

Velamentous cord insertion is one of the abnormal cord insertion types, in which the vessels run through the amnion and chorion, unprotected by Wharton’s jelly, before reaching the placental plate [44,59]. This abnormality is present in 1.5% of singleton pregnancies [59]. It is associated with several unfavorable perinatal outcomes, such as fetal growth restriction, placenta praevia, placental abruption, fetal heart abnormalities during labor, emergency Cesarean section, preterm labor, low Apgar scores, admission to the NICU and neonatal death, as the velamentous vessels can be compressed during uterine contractions and fetal movements [43,60]. The risk factors associated with this condition include bilobate/succenturiate placenta, ART, multiple gestation and a prior history of abnormal placental cord insertion [59] (Figure 18, Figure 19, Figure 20, Figure 21 and Figure 22).

#### 3.2.4. Marginal Umbilical Cord Insertion

**Figure 23:**
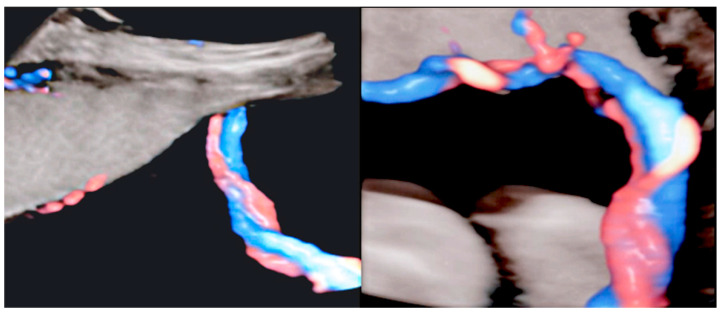
Marginal insertion of the umbilical cord using HD-Flow™ Doppler ultrasound.

Marginal umbilical cord insertion is another anomaly, in which the umbilical cord is inserted into the placental margin within 2.0 cm [44,60]. It is present in 6.3% of singleton pregnancies and presents much lower chances of complications than velamentous cord insertion [60] (Figure 23).

#### 3.2.5. Furcate Umbilical Cord Insertion

**Figure 24:**
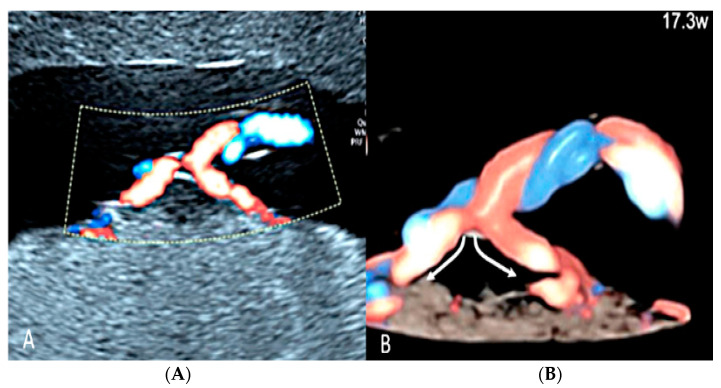
Furcate umbilical cord insertion using two-dimensional ultrasound (**A**) and three-dimensional ultrasound (**B**) with HD-Flow™ Doppler: the dichotomic direction of the umbilical blood flow is clearly demonstrated at 17.3 week’s gestation (arrows).

Furcate umbilical cord insertion is a condition where the umbilical vessels separate before they insert into the placental plate and lose their Wharton’s jelly [59,60]. It is present in 0.1% of pregnancies and is considered a variant not well distinguished from velamentous cord insertion [59,60] (Figure 24).

#### 3.2.6. Umbilical Cord Entanglement

**Figure 25:**
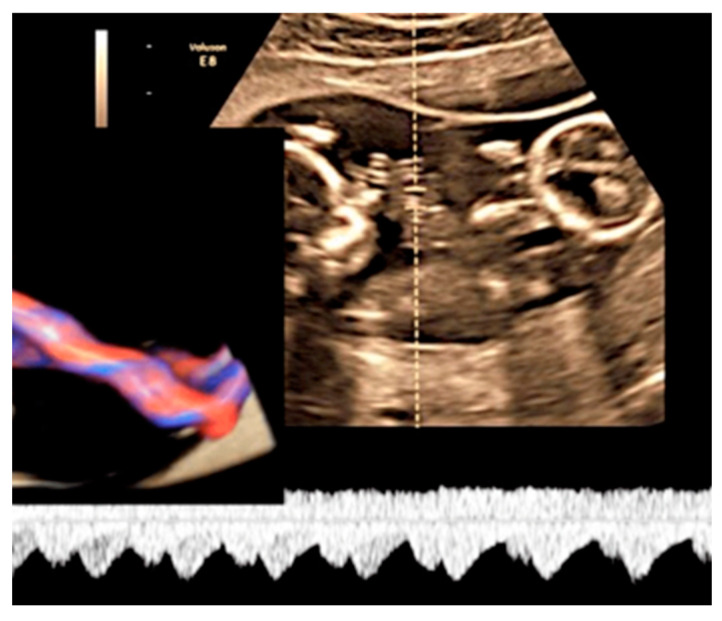
MCMA twin pregnancy. Cord entanglement can also be detected with the “galloping horse sign” that involves identifying two distinct waves signals with different heart rates obtained by sampling an apparently unique umbilical cord with pulse wave (PW) spectral Doppler ultrasound.

Umbilical cord entanglement is a condition exclusively of monoamniotic twin pregnancies and occurs because both umbilical cords insert close to each other in the single placenta [47]. The entangled cords can cause vascular damage for one or both fetuses and lead to fetal demise [47]. Diagnosis can be made through the visualization of a branch pattern on color Doppler ultrasound at the level of the entanglement, and an end systolic notch seen on the umbilical artery waveform reflects vascular compression or narrowing [47,61] (Figure 25).

#### 3.2.7. Umbilical Cord Hemangioma 

**Figure 26:**
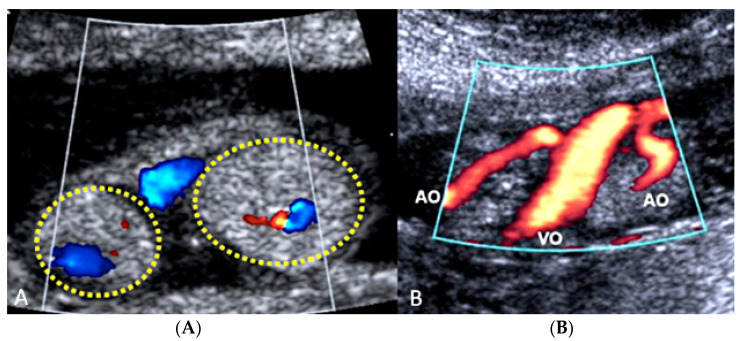
Sonographic evaluation of umbilical cord hemangioma. (Legend: AO: umbilical artery; VO: umbilical vein). Umbilical cord hemangioma or angiomyxoma is usually located in the terminal portion of the cord on the placental side. Two-dimensional color (**A**) and power Doppler ultrasound (**B**) may detect a fusiform or saccular swelling of the umbilical cord. Umbilical arteries are surrounded by a particularly echogenic material arranged as a sheath of the same vessels. The mechanical compression of the umbilical circulation may be determined by an enlargement of the mass and stenosis of the umbilical vessels also caused by the intraluminal proliferation of the hemangioma. Blood flow reduction can be abruptly compromised through the twisting of the cord favored by the mass and by the presence of pseudocysts. An accurate follow-up is mandatory.

**Figure 27:**
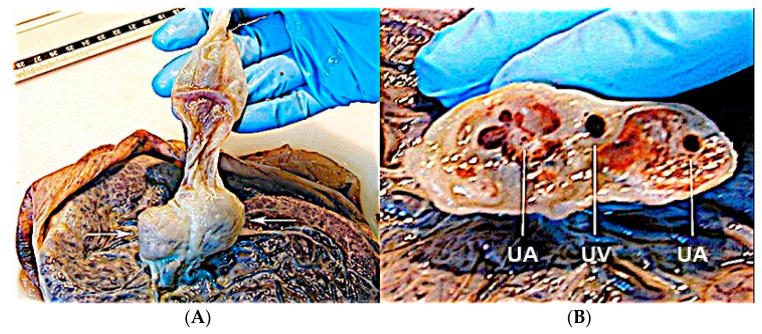
Pathologic correlation. Umbilical cord hemangioma at the fetal face of the placenta (**A**) and the axial section of the umbilical cord (**B**) showing a large hematoma in one of the two umbilical artery (UA: umbilical artery; UV: umbilical vein). Evident increase in the diameter of the cord due to swelling and myxoid-edematous degeneration. The section of the cord shows the two umbilical arteries surrounded by thick angiomatous tissue.

Umbilical cord hemangioma is the most frequently reported cord tumor and is usually located at the placental insertion site [47]. It has been associated with congenital anomalies and increased perinatal mortality [62], mainly due to impaired umbilical circulation owing to tumor proliferation, vascular compression, intravascular thrombosis or fetal hemorrhage due to ruptured vessels [63]. Sonographic evaluation shows a hyperechogenic mass within the umbilical cord, which may also appear edematous, and it has differential diagnosis with hematomas and teratomas, placental masses and abdominal wall defects [62,63] (Figure 26 and Figure 27).

#### 3.2.8. Umbilical Cord Hematoma 

**Figure 28:**
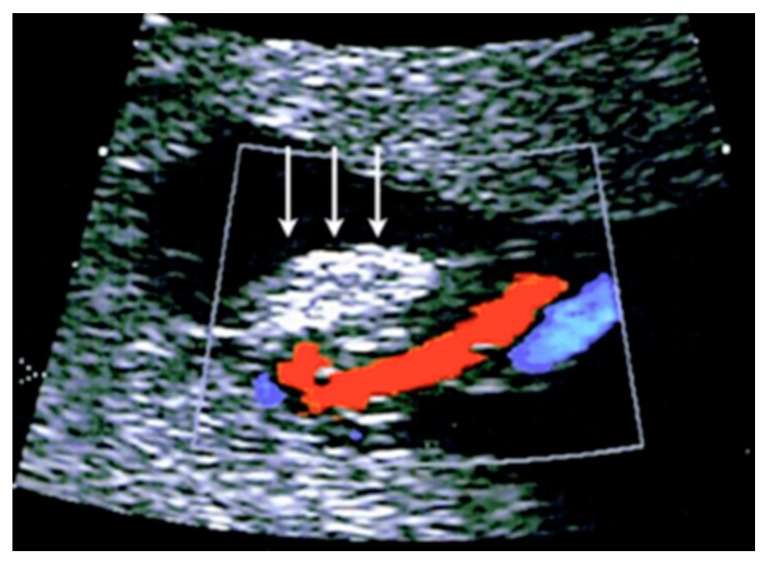
Transient umbilical cord hematoma in the cord insertion area on the placenta after intra uterine fetal transfusion (IUFT). Note the absence of blood flow on color Doppler ultrasound assessment (arrows).

Umbilical cord hematomas are rare events that occur in 1:5,500 pregnancies and mostly take place due to the rupture of the umbilical vein rather than the umbilical arteries [64]. They are associated with oligohydramnios and second-trimester amniocentesis, and the compression of the umbilical vessels induced by a cord hematoma can cause fetal hypoxia or intrauterine death in up to 40% of cases [64]. At ultrasound, it appears as a focal mass on the cord without blood flow on color Doppler ultrasound [47,63] (Figure 28).

#### 3.2.9. Umbilical Cord Cyst

Umbilical cord cysts can be classified as true cysts and pseudocysts. True cysts are present in 3.4% of first trimester pregnancies and 20% of those persist into the second trimester [65]. They are usually located close to fetal insertion of the cord and vary between 4–6 mm in diameter. They are associated with a 20% increase in the prevalence of aneuploidy and structural anomalies [65]. 

**Figure 29:**
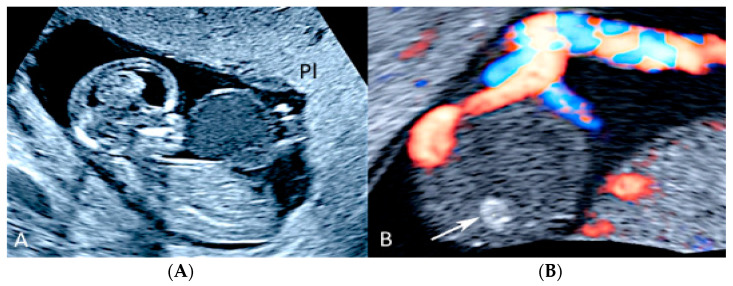
Pregnancy at 12 week’s gestation. Two-dimensional ultrasound: sagittal scan of the fetus showing a cyst filled with finely corpuscular material at the level of the cord insertion on fetal abdomen (**A**)**.** Color Doppler ultrasound highlights the cord’s splayed vessels due to a hemorrhagic cyst with a clot inside it (arrow) (**B**). (Legend: Pl: placenta).

Pseudocysts (Wharton’s jelly cysts) are more frequent than true cysts and tend to be located close to the fetal heart insertion site of the umbilical cord [65]. They are formed by localized edema and the liquefaction of the Wharton’s jelly and have no epithelial lining [65]. These cysts are associated with fetal trisomy and other congenital anomalies, including exomphalos, vertebral defects, imperforate anus and tracheoesophageal fistula [65]. Sonographic assessment shows a cystic mass along the cord without internal flow on color Doppler ultrasound [47]. If another fetal anomaly is seen together with umbilical cord cyst, karyotyping is indicated [65] (Figure 29).

#### 3.2.10. Single Umbilical Artery

**Figure 30:**
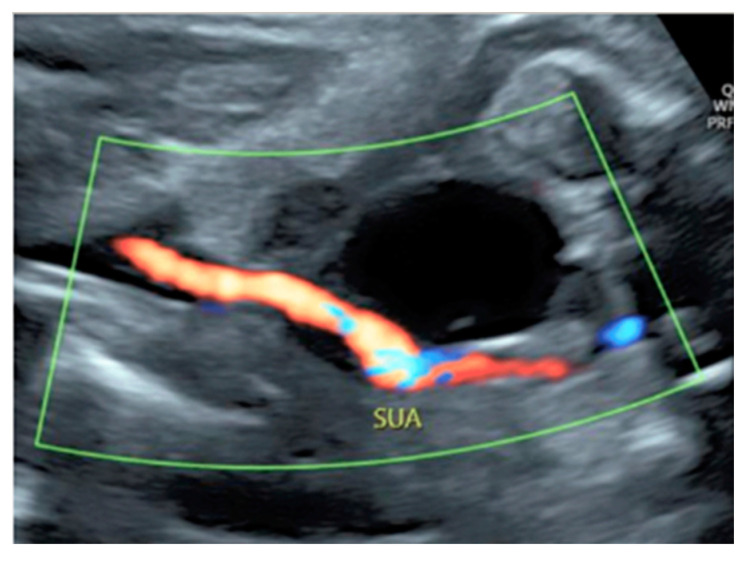
Doppler ultrasound showing a single umbilical artery (SUA) coursing along the bladder.

**Figure 31:**
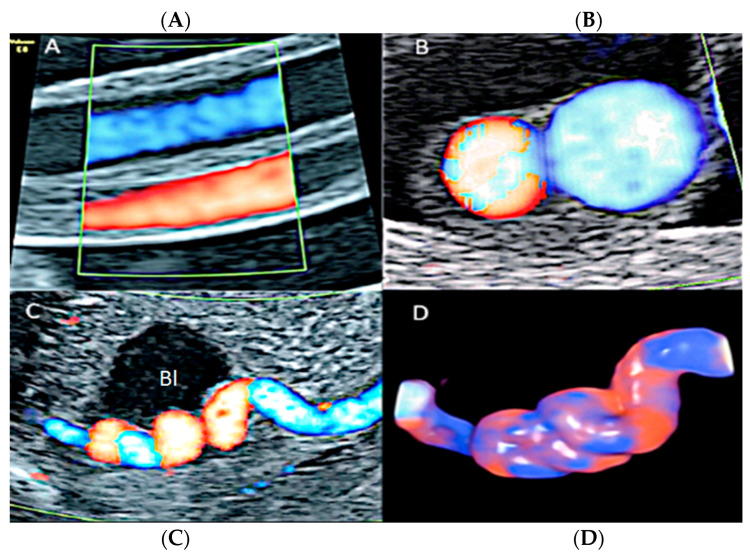
Pregnancy at 22 week’s gestation. Color Doppler (**A**–**C**) and four-dimensional STIC with color Doppler ultrasound in “glass-body” mode (**D**). Longitudinal and axial scan of the cord (**A**–**C**) demonstrate the presence of a single umbilical artery (SUA). SUA shows a particular paravesical kinking (Bl: bladder).

A single umbilical artery (SUA) has a prevalence of 1.0% of all pregnancies, with greater frequency of an absent left umbilical artery than absent right umbilical artery [47]. Fetuses with isolated SUA have a general favorable in utero prognosis while those with other associated congenital anomalies are at higher risk of chromosomal abnormalities, pregnancy complication, mortality and morbidity in the neonatal period [47,66,67]. Sonographic diagnosis can be made on an axial view of the umbilical cord, with only two vessels seen on grey-scale, or by visualizing only one artery next to the bladder on the fetal pelvis using color Doppler ultrasound [47] (Figure 30 and Figure 31).

#### 3.2.11. Umbilical Cord Varix

**Figure 32:**
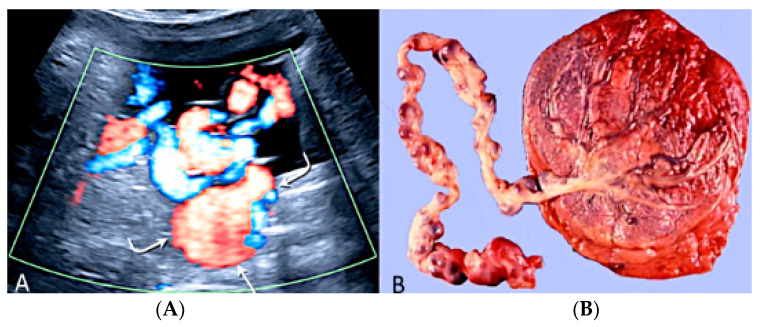
Doppler ultrasound (**A**) demonstrating an umbilical cord varix (curved arrows) with pathology confirmation after birth (**B**).

Umbilical cord varix is a rare condition in which there is a focal dilatation larger than 9.0 mm in the intrahepatic or extrahepatic portion of the umbilical vein [47,68]. It is associated with a 5.8% risk of chromosomal aneuploidy, especially trisomy 21, and a 19% risk of additional ultrasound anomalies [68]. At ultrasound examination, a cystic structure is seen in the fetal abdomen, showing differential diagnosis to normal fetal gallbladder and fetal stomach mesenteric or omental cysts and umbilical artery aneurisms [47]. Doppler ultrasound evaluation is essential for a correct diagnosis [47] (Figure 32).

#### 3.2.12. Umbilical Vessel Thrombosis

**Figure 33:**
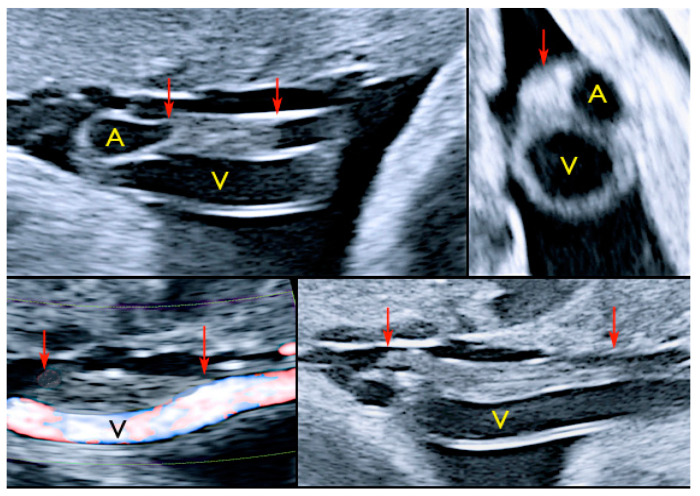
Pregnancy at 32, 34 and 35 week’s gestation. Two-dimensional ultrasound in longitudinal and axial scan of the umbilical cord. A moderate echogenicity corresponds to segmental thrombosis of an umbilical artery. The thrombosis zone is indicated by red arrows and the progressive extension is further delimited by arrows (Legend: A: umbilical artery; V: umbilical vein).

Risk factors for umbilical vessel thrombosis include the hypercoiling of the umbilical cord and deficient Wharton’s jelly [69]. Umbilical artery thrombosis is a rare event and is associated with intrauterine growth restriction, fetal organ infarcts and stillbirth [69]. Umbilical vein thrombosis is also a rare event and is associated with worse perinatal outcomes when compared to umbilical artery thrombosis, as there is only one vein in the umbilical cord to deliver oxygenated blood from the mother to the fetus [69]. Generally, the diagnosis is made after birth, but sonographic diagnosis is possible by visualizing an echogenic core representing the thrombus, associated with turbulent flow on color Doppler with a dilated umbilical segment, when the vein is not completely occluded, or by not finding two blood flows within the umbilical arteries [69] (Figure 33).

#### 3.2.13. Umbilical Cord Vessel Aneurysm

**Figure 34:**
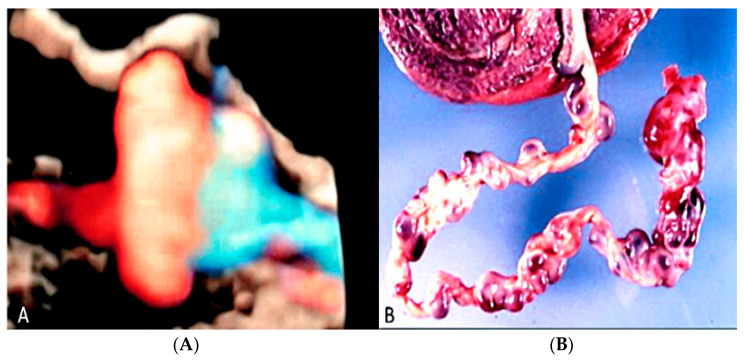
Sonographic view of umbilical cord vessel aneurism with color Doppler ultrasound (**A**). Gross anatomy: umbilical cord after delivery confirmed presence of multiples aneurisms (**B**).

Umbilical cord vessel aneurysm is a rare obstetrical condition due to a congenital thinning of the vessel wall [69]. It is usually associated with a single umbilical artery (SUA) and other adverse perinatal outcomes, such as chromosomal aneuploidies, intrauterine growth restriction, cardiac abnormalities, fetal structural anomalies and stillbirth [47,69]. Sonographic diagnosis can be made by visualization of a focal dilatation of the umbilical cord associated with blood flow detected by color Doppler ultrasound [69] (Figure 34).

## 4. Discussion

Although rare, placental and umbilical cord pathologies have a significant association with adverse perinatal and obstetrical outcomes. A proper obstetric evaluation should analyze not only fetal characteristics, but also placental patterns, such as location, thickness, morphology, presence of multiples lobes, presence of membranes in multiple gestation, in addition to correlation with maternal symptoms such as pain or vaginal bleeding [70,71]. Under the same reasoning, an ultrasound screening should evaluate aspects of the umbilical cord that can lead to unfavorable outcomes, such as vasa preavia, velamentous cord insertions and the number of umbilical arteries [72]. An initial assessment should be made during the first trimester scan for both placental position and cord insertion, while a thorough evaluation should be performed in the second trimester ultrasound for a correct diagnose [69,70,71,72]. During third trimester, imaging follow-up should be done for a better decision on the moment and way of delivery, improving the chances of mother and newborn well-being. 

Although placental and umbilical cord can be diagnosed using 2D ultrasound, the release of new 3D technology has further enhances the quality of the imaging enabling sonographers and clinicians to reach the correct prenatal diagnosis and plan the appropriate antenatal and postnatal management. Not only a technological ultrasound improvement has been produced in term of surface imaging such as softwares as “glass-body”, Silhoutte™, Cristal Vue™ or other available commercially techniques but a significant improvement has been seen in Doppler ultrasound analysis with tools like the iFlow™ or Radiantflow™ that are extremely powerful for the vascular branching of low velocity vessels and flow like that of the placenta and umbilical cord.

## Data Availability

The data presented in this study are available on request from the Corresponding author.
